# Supplementary material for: Prognostic Relevance of NPM1 and FLT3 Mutations in Acute Myeloid Leukaemia, Longterm Follow-Up—A Single Center Experience
Source: Cancers (Basel). 2022 Sep 28;14(19):4716. doi: 10.3390/cancers14194716 (PMC9562865; doi:10.3390/cancers14194716)
Supplement: Supplementary file 1 [file cancers-14-04716-s001.zip › cancers-1905985-supplementary.pdf]

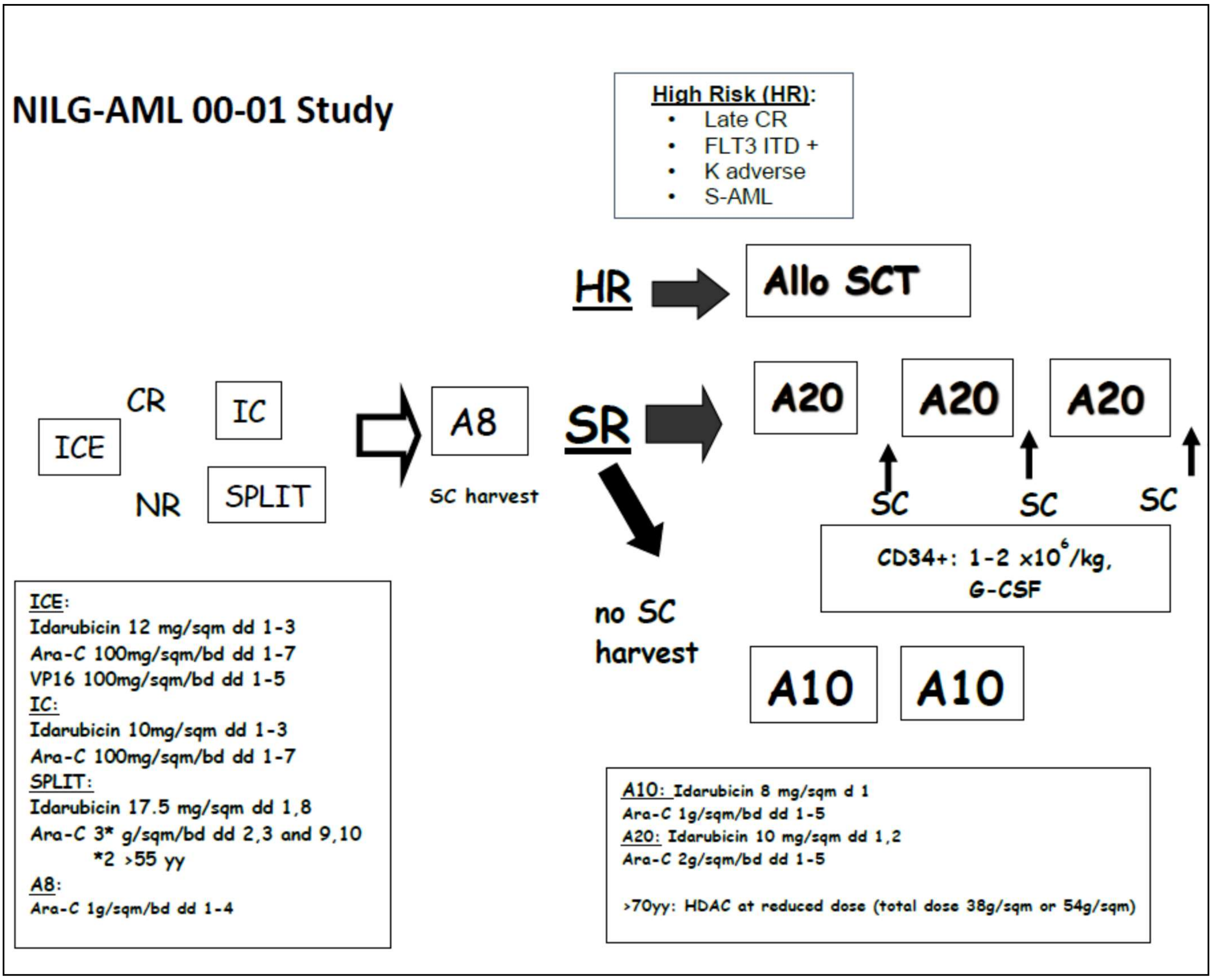

Figure S1. Treatment Plan.

Table S1. Characteristics of 366 AML patients, incidence of molecular mutations (*NPM1* and *FLT3*).

**Overall**		Number (%)
		366
**Sex**	male	203 (55.5)
	female	163 (44.5)
NPM1	mutated	132/327 (40.4)
	unmutated	195/327 (59.6)
	not available	39/366 (10.6)
FLT3	mutated	98/352 (27.8)
***FLT3*-ITD**	mutated	65/352 (18.5)
	unmutated	287/352 (81.5)
	not available	14/366 (3.8)
***FLT3*-TKD**	mutated	33/316 (10.4)
	unmutated	28/316 (8.9)
	not available	50/366 (13.6)
***NPM1*/*FLT3*-ITD**	*NPM1* m/*FLT3*-ITD wt	73/138 (52.9)
	NPM1 m/*FLT3*-ITD m	38/138 (27.5)
	NPM1 wt/*FLT3*-ITD m	27/138 (18.6)
**Karyotype Risk Classification***	favorable	38 (10.4)
	intermediate	265 (72.4)
	adverse	59 (16.1)
	not available	4 (1.1)

Abbreviations: wt: wild-type; m: mutated.

Notes: *according to MRC [22].
